# Effective management of depression in primary care: a review of the literature

**DOI:** 10.3399/bjgpopen17X101025

**Published:** 2017-06-28

**Authors:** Bruce Arroll, Fiona Moir, Tony Kendrick

**Affiliations:** 1 Elaine Gurr Chair of General Practice and Primary Health Care, and Director of the Goodfellow Unit, Department of General Practice and Primary Care, University of Auckland, Auckland, New Zealand; 2 Senior Lecturer, Department of General Practice and Primary Care, University of Auckland, Auckland, New Zealand; 3 Professor of Primary Care, Primary Care and Population Sciences, University of Southampton, Southampton, UK

**Keywords:** depression, review, primary care

## What is depression: a biochemical disorder or a social construct or both?

There is an argument that clinicians are medicalising normal human suffering in many of their patients. In his, book *Beyond Depression*, Chris Dowrick says '… our culture is imbued with an expectation of happiness, and that in consequence we tend to see negative emotional experiences as aberrant and deviant'.^1^ He proposes 'an approach to patients which emphasises listening and understanding rather than diagnosis and prescription.' Many GPs will be sympathetic to these views, but nevertheless have to respond to patients’ needs while wrestling with time pressure and limited access to non-drug therapies. The temptation is to follow the biomedical model of diagnosis and treatment.

## Formal definitions of depression

Depressive symptoms range on a continuum from everyday sadness to suicidal despair, and a challenge for practitioners is deciding at what level to intervene. A clinical definition of depression is therefore necessary to inform clinical decisions, enable research to be conducted, and justify insurance payments. The most widely accepted definition of depression comes from the 5th edition of the *Diagnostic and Statistical Manual of Mental Disorders* (DSM-5) published by the American Psychiatric Association. The DSM-5 outlines three degrees of severity (mild, moderate, and severe) based on a required number of symptoms and the degree of functional impairment. The presence or absence of psychotic features, such as hallucinations or paranoia, help determine specific treatments.

## Other ways of measuring depression severity to facilitate choice of treatment

The National Institute of Health and Care Excellence (NICE) recommends the use of depression symptom inventories to assess severity and to monitor improvement.^2^ Commonly used inventories include the patient health questionnaire 9 (PHQ-9) and the Hospital Anxiety and Depression Scale (HADS). While they are not considered diagnostic tools they have been validated against gold standard clinical interviews and can be very handy for use in a busy primary care clinic. Their main use is in giving a measure of severity and in monitoring the progress of treatment. Table 1 shows the ranges for each inventory.

**Table 1.** Range of depression scores versus severity for PHQ-9 and HADS

Severity	PHQ-9 (maximum 27)	HADs (maximum 21)
Minimal	1–4	0–7
Mild	5–9	8–10
Moderate	10–14	0
Moderate–severe	15–19	≥11
Severe	≥20	0

## Detecting bipolar disorder

A recent study reported that about 10% of patients prescribed antidepressant medication actually have undetected bipolar disorder. The authors recommended 'When seeing patients with depression or anxiety disorder, particularly when they are young or not doing well, clinicians should review the life history for evidence of unrecognised bipolar disorder.'^3^ Therefore if the patient has had periods of inappropriately elevated mood, overactivity, or disinhibited behaviour which have lasted for 4 days or more, consider referral for a specialist mental health assessment.

## Complex situations

More complex situations require careful consideration. For instance, the literature states that personality traits such as ‘neuroticism’ increase the risk of depression when faced with stressful life events,^4^ and that it is common for people with a personality disorder to have coexisting depression,^5^ Practitioners will be aware of the challenges this can bring to the therapeutic relationship and the likelihood of a successful collaborative approach. However, there may be factors within the patient, the practitioner, the doctor–patient relationship, the health system, or other external circumstances which are compounding the issue, and it can be valuable to reflect on each of these components if the practitioner is willing. The diagnosis of 'personality disorder' can be a useful signal to take a more reflective approach and remember to focus on the main message without getting side-tracked, and to empathise and separate the person from the behaviour. An overemphasis on personality may not perhaps be helpful, especially if patients have not had trials of psychological or drug therapy for their longstanding depressive symptoms. An assessment of someone's personality while they are depressed may provide misleading information, and is something that should be re-evaluated after their depression has been treated. Therefore, steering away from labels and focusing on skilful communication to optimise the relationship may give the best chance of a successful outcome.

Another challenging example is a patient with depression and a co-existing substance use disorder, of which alcohol is the most common. There are some broad principles which can be applied, such as being careful to establish a clear history of symptoms and events which may involve taking a collateral history from family members, using the consultation to also focus on their physical health, and being aware that they are more likely to be lost to follow up. Possible interactions between the person's antidepressants and other medications or illegal drugs should be considered, along with the fact that they may require a different medication dose to achieve a therapeutic effect. Antidepressant choice may be influenced by the person's ability to follow instructions, and an assessment of suicidality. A multi-agency approach may be beneficial.^6^

## Depression or anxiety: the emergence of transdiagnostic approaches

There is considerable overlap in symptoms between depression and anxiety disorders. The MaGPIe study from New Zealand found that 18.1% of primary care patients met the criteria for depression over the past 12 months, but 56% of them had a co-existing DSM IV level anxiety disorder, and 20% had a substance use and dependence disorder.^7^ The first line drug treatments are identical for both depression and anxiety, as are the psychological therapies and ‘transdiagnostic’ approaches to treatment and diagnosis that are emerging.^8,9^ Multiple cognitive behavioural therapy (CBT) protocols have been developed to deal with the many subtypes of anxiety, but transdiagnostic treatments are based on the notion that these various protocols contain important but overlapping treatment components that can be distilled into a single treatment to address the symptoms across all of the disorders at once.^9^ Transdiagnostic terminology is used explicitly for diagnosis in one of the so-called ‘third wave’ cognitive behavioural therapies, known as acceptance and commitment therapy (ACT). In ACT, patients are described as being 'stuck'.^10^ Those who use ACT in primary care spend less time on diagnosis in order to give more time to therapy.^10^

## The NICE stepped-care model

NICE's a stepped-care model for the detection and management of depression is as follows:

- Step 1 — Assessment and initial management; first visit.
- Step 2 — Persistent subthreshold depressive symptoms; mild-to-moderate depression.
- Step 3 — Persisting symptoms after step 2; interventions; moderate and severe depression.
- Step 4 — Severe and complex depression.

### Step 1 — Assessment and initial management; first visit

Detecting depression at a single GP visit is difficult as 70–90% of patients with depression and anxiety present with symptoms of somatic illness.^11^ Psychosocial issues, which may be the main reason for the visit, are likely to be left until the last minute and mentioned as the patient is about to leave (hence the name ‘exit’ or ‘doorknob’ comment).^12^ The GP often has to make a split second decision on whether to explore the issues at that visit and run late or re-book for another time. There may only be enough time to ask the two questions NICE recommends for case finding, specifically: 'during the last month, have you often been bothered by feeling down, depressed, or hopeless?' And 'during the last month, have you often been bothered by having little interest or pleasure in doing things?' The sensitivity of these questions is high in primary care which means that if a patients answers 'no' to both questions they are highly unlikely to have a major depression.^13^ If the answer to either question is 'yes', further questioning is needed and the PHQ-9 is useful as it can give a level of depression which corresponds to a recommended treatment action (Box 1). Where time is short, an option is to give the PHQ-9 form to the patient to complete at home and book an appointment in the near future.

**Box 1.** PHQ-9 scores at diagnosis and proposed treatment actions^17^

PHQ-9 score	Depression severity	Proposed treatment actions
0–4	None–minimal	None
5–9	Mild	Watchful waiting; repeat PHQ-9 at follow-up
10–14	Moderate	Psychological treatment and follow-up/antidepressants
15–19	Moderate–severe	Antidepressants and psychological treatment
20–27	Severe	Referral to mental health

NICE recommends seeing patients 2 weeks after starting antidepressants. There is no evidence base for choosing such a time interval but we are influenced by the comments of a UK GP in the 'Insider’s guide to depression' where she says '... see us frequently at first, a week is a long time in a Dali landscape. Three weeks are almost unimaginable'.^14^ One option, if a subsequent visit must be delayed, is to offer a nurse phone call. A randomised controlled trial with patients who were all on antidepressants found an number needed to treat (NNT) of 5 to get an improvement making it more effective than antidepressants for mild or moderate-severe depression.^15,16^

#### 'Should antidepressants be started at the first visit?'

This specific question has not been tested in clinical trials, but from our experience patients with high PHQ-9 scores at the first visit will invariably have lower ones at the next. This may be a combination of the therapeutic action of talking with the GP including normalisation, reframing, reassurance, and the offering of hope, as well as regression to the mean. Patients can have high and very high PHQ-9 scores and not have a major depression (Table 2) and the PHQ-9 should be considered as a measure of distress rather than a diagnostic tool *per se*. We concur with NICE that the choice of intervention will be influenced by the duration of the episode, the trajectory, and experience from previous courses of depression and response to treatment along with the patient’s treatment preferences and priorities. There are many other factors to consider, such as the skill level and interest of the GP in mental health, their ability to communicate about sensitive topics, the patient’s culture, and level of perceived stigma of different treatments, along with practicalities, such as time, financial stressors, transport, access to computers, and availability of non-drug resources.

**Table 2.** Mismatch between PHQ-9 symptom questionnaire scores and psychiatric diagnoses.

Severity on PHQ-9, *N *= 2642	Depressed on DSM IV, *N* = 583 (% all patient)	Not depressed on DSM IV, *N *= 2059 (% all patient)
Mild 5–9	156 (6)	402 (15.2)
Moderate 10–14	102 (3.9)	102 (3.9)
Moderate-severe 15–19	66 (2.5)	24 (0.9)
Severe 20–27	37 (1.4)	10 (0.4)

#### Are antidepressants effective for mild-to-moderate depression?

**Figure 1.**
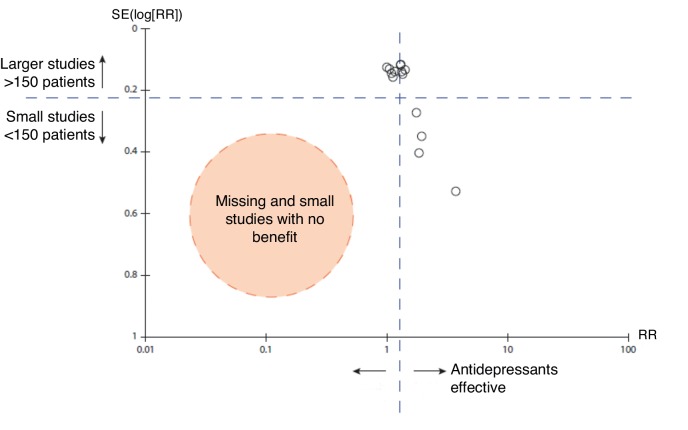
Funnel plot of SSRI and TCA versus placebo from primary care antidepressant versus placebo review.^18^

The majority of patients in primary care with depression are in the mild (6%) or moderate levels of severity (3.9%) and 3.9% are in the moderate-severe to severe range (Table 2). Patient level meta-analysis found that the NNT for milder-to-moderate depression was 16, 11 for severe, and for very severe 4.^16^ The median control event rate (the placebo response) is about 47% in primary care trials of antidepressants.^18^ An NNT of 16 represents a 6% gain in terms of a better outcome. Thus, in a randomised controlled trial of patients in the mild-to-moderate range 53% would get better with an active medication and 47% would get better with placebo. The authors of this patient level meta-analysis found that true drug effects (for instance an advantage of antidepressant medication over placebo) were non-existent with mild-to-moderate and even severe baseline symptoms but were large for those with very severe symptoms. This was confirmed by Kirsch *et al* and they reported a lower placebo rate in the very severe group in a review where all studies registered with the FDA were included.^19^ A systematic review of antidepressants in primary care found that medication appeared to be effective for depression but there was evidence of publication bias for both selective-serotonin reuptake inhibitors (SSRIs) and tricyclic antidepressants (TCAs).^18^ Indeed when the SSRIs and TCAs were pooled in the same analysis there were no small studies (<150 participants) with negative results (Figure 1), yet there were four small studies with positive results. In this primary care review 7 of 11 studies included patients with HAM-D scores >18 which is considered the severe range.^18^

NICE therefore suggests not using antidepressants routinely to treat persistent subthreshold depressive symptoms or mild depression because the risk–benefit ratio is poor. We would add that as the NNT for benefit is much greater than 1, most people do not benefit from antidepressants, and that improvements are more likely to be a placebo response rather than a medication response. For those in the mild-to-moderate range, who feel they have benefited from taking an antidepressant, are eight times more likely to have had a placebo response than a true medication response. Patients are likely to give medication the credit for getting better and may medicalise future episodes of low mood by requesting antidepressants. There is also the issue of having a withdrawal syndrome when trying to stop these medications which can in turn lead to long-term use of antidepressants. NICE advises avoiding drug treatment unless there is a past history of moderate or severe depression that persists after other interventions, or subthreshold symptoms that have been present for a long period typically at least 2 years.

### Step 2 — Persistent subthreshold depressive symptoms; mild-to-moderate depression

NICE recommends low intensity psychosocial interventions such as sleep hygiene, individualised self-help principles of CBT, computerised CBT with or without a facilitator, problem-solving therapy, behavioural activation, and physical activity (recommended in groups) for the initial management of mild-to-moderate depression or sub-threshold persisting symptoms. Hallgren *et al* write (based on their 2016 randomised controlled trial) that exercise is beneficial for depression even when it is light (yoga and stretching, for example) as opposed to moderate and vigorous and when conducted once per week.^20^ They also mention the cardiovascular and metabolic benefits for patients with depression.

NICE recommends group-based CBT for patients in this category presumably based on cost and efficiency. They advise against St John’s Wort even though it has been shown to be effective due to variation in the nature of preparations, uncertainty about appropriate doses, and potential serious interactions with other drugs. Other potential non-drug therapies are acceptance, as is commitment therapy and mindfulness CBT, transcranial magnetic/direct current stimulation (still experimental), interpersonal psychotherapy, psychodynamic therapies, and psychotherapy.^21^ Some therapists suggest that it is important to initiate some therapeutic intervention at the first visit as many patients do not return for follow-up visits.^12^

### Step 3 — Persisting symptoms after step 2; interventions; moderate and severe depression

NICE suggests for patients with persistent subthreshold symptoms or mild-to-moderate depression who have not benefitted from a low-intensity psychosocial intervention to discuss the relative merits of medication versus CBT. Then offer an antidepressant (normally an SSRI) or high intensity psychological intervention with either CBT, interpersonal therapy (IPT), behavioural activation, or behavioural couples therapy where appropriate. NICE recommends combination antidepressant medication and high-intensity CBT or IPT for moderate-to-severe depression although there is little empirical evidence to support combination therapy other than one trial where CBT was added to patients on medication with treatment of resistant depression in primary care, with an NNT of 4 to achieve a 50% reduction in baseline scores.^22^ NICE suggests avoiding the antidepressant medicines fluoxetine, fluvoxamine, and paroxetine in the first instance as they have a higher propensity for drug interactions. NICE also suggests avoiding venlafaxine and tricyclic antidepressants (TCAs) as they have a greater risk of death in overdose. Sertraline can be used in patients with heart disease and in pregnancy and is one of the most effective of the newer antidepressants.^23^ Mirtazapine, an alpha antagonist, is an effective antidepressant and has the benefit of having a sedative effect and fewer gastrointestinal side effects, but the potential disadvantage of weight gain.^23^

### Step 4 — Complex and severe depression

Referral to specialist mental health services is necessary for individuals who are at a significant risk of self-harm, have psychotic symptoms, require complex multiprofessional care, or where an expert opinion on treatment and management is needed.

## Relapse prevention

A systematic review examining 54 trials of relapse prevention for depression found conclusive evidence that the continuation of antidepressants was effective in preventing recurrent depression (odds ratio = 0.35; 95% confidence interval = 0.32 to 0.39).^24^ However, nearly all of the studies included were based in secondary care and are probably not representative of primary care patients. They also failed in a number of trials to distinguish between withdrawal symptoms, which are usually temporary, and true relapse. More research on the need for long-term antidepressants to prevent relapse is underway in primary care.

Mindfulness-based cognitive therapy (MBCT) is a non-drug option for preventing relapses of depression. Kuyken and colleagues’ group MBCT helped 70% of participants stop antidepressants, without increasing relapse. The therapy is intensive however, and the skills required in short supply. Group treatment could be provided through the IAPT programme in the UK, but many services are already stretched. The study was limited to patients in equipoise over psychological versus antidepressant treatment, which may not apply to many fearful of relapse, and many will prefer individual rather than group therapy.^25^

## Conclusion

Psychological therapies should usually be first choice for most patients with mild-to-moderate depression in primary care, and drug treatment should only be used for moderate-to-severe depression in the first instance, or for mild-to-moderate depression when active monitoring and other treatments have failed.
